# Prevalence of acute respiratory infections among children in India: Regional inequalities and risk factors

**DOI:** 10.1007/s10995-022-03424-3

**Published:** 2022-04-18

**Authors:** Md Masud Hasan, Kamal Kumar Saha, Rossita Mohamad Yunus, Khorshed Alam

**Affiliations:** 1 Research School of Social Sciences, ANU College of Arts & Social Sciences, The Australian National University, Canberra, ACT 2601 Australia; 2 College of Engineering and Technology, CQUniversity, Melbourne, Australia; 3 Institute of Mathematical Sciences, Faculty of Science, University of Malaya, 50603 Kuala Lumpur, Malaysia; 4 School of Business, Faculty of Business, Education, Law & Arts, University of Southern Queensland, Toowoomba, QLD 4350 Australia

**Keywords:** Acute respiratory infection, India, Risk factors, Regional inequalities

## Abstract

**Aim:**

The high incidence of acute respiratory infection (ARI)-related morbidity and mortality is a major public health concern in developing countries. This study aimed to quantify regional inequalities and the degree of association between childhood ARI and background factors.

**Methods:**

This study utilised information of 238 945 children aged below five years extracted from the Fourth Indian National Family Health Survey conducted in 2015–16. Inter-state and regional inequality in the prevalence of ARI were quantified and presented using a map of India and forest plot. The association of background characteristics and ARI was quantified using bivariate and multivariable binary logistic regression models.

**Results:**

Significant inequalities in the prevalence of childhood ARI were observed across the six regions of India. Considering the children from north-east region as a reference, those from north, central and east regions were 0.68, 1.02 and 0.57 times more likely to suffer from ARI. Comorbidity, sex, age and nutritional status of children were significantly associated with the prevalence of ARI.

**Conclusions:**

ARI remains a significant public health concern among Indian children. The results of this study showed that significant regional disparities in the prevalence of ARI exist in India. This study adds value to the better understanding of inequality patterns and quantifies within- and intra-region inequalities in the prevalence of ARI in India.

## Introduction

The Sustainable Development Goals (SDGs) set the target to end deaths from preventable diseases among new-borns and children under five years old by 2030 (Dahan & Gelb, 2015). Nevertheless, in 2017, an estimated 5.5 million children belonging to this age group died from preventable diseases (Hug et al., 2018). One of the impediments to achieve this goal adopted by all United Nations member states is the high prevalence of acute respiratory infection (ARI), a leading cause of childhood morbidity and mortality. In 2016, from 68.06 million episodes, an estimated 652 572 children aged below five years died because of lower respiratory infections (Troeger et al., 2018; Walker et al., 2013) reported that the incidence of severe ARI is the highest in Southeast Asian and African regions. India is one of the 15 highest burdened countries in terms of total pneumonia episodes and related childhood mortality. In India, around 400 000 children aged below five years die every year from ARI-related diseases. The figure accounts for 13–16% of all child deaths among paediatric hospital admissions (Jain et al., 2001; Vashishtha, 2010). As a cause of approximately one-fourth of global annual deaths of children aged below five years, ARI is a significant public health concern in India (Mathew et al., 2011).

The incidence of ARI is associated with a multitude of factors related to the demographics of children, socioeconomic background of their parents and place of residence and the household environment where they grow up. In Bangladesh and India, a relatively high incidence of ARI episodes has been observed among the young, non-exclusively breastfed, anaemic children and those with low birth weight (Budge et al., 2014; Hasan & Richardson, 2017; Prajapati et al., 2011; Sheikh Quyoom Hussain et al., 2014). As a primary career, the socio-cultural, economic and educational backgrounds of the mother are associated with the incidence of childhood ARI. Contemporary literature has reported the significant association between maternal illiteracy, knowledge base and access to mass media and incidence of ARI (Kamal et al., 2015; Nirmolia et al., 2018; Ramani et al., 2016; Sharma et al., 2013; Tazinya et al., 2018). However, the association between maternal education and the prevalence of ARI was insignificant in other studies (Asghar et al., 2017; Goel et al., 2012). The caste, religion and tribal status of a mother as a proxy to their social class showed significant association with the incidence of ARI amongst Indian children (Prakash, 2014). Unclean fuel sources, including biomass and charcoal, have been considered major determinants of childhood ARI (Mathew et al., 2011). Children living in large-size families and overcrowded houses are likely to suffer from ARI (Prajapati et al., 2011). Living at high altitude influences the prevalence of childhood ARI, and an altitude above 2500 m is a modest predictor for respiratory syncytial virus infection in the USA (Choudhuri et al., 2006). Rural–urban gap evidently affects the incidence of childhood ARI (Mathew et al., 2011). However, the unequal distribution of the prevalence of ARI episodes across the states and regions of India has not been completely explored. Any effort to improve the child mortality target of SDGs would largely depend on the progress made in India given that it contributes to one-fifth of global live births and more than a quarter of neonatal mortality (Sankar et al., 2016). Thus, the prevalence and determinants of ARI must be understood at the regional level because any burden estimates at the national level may camouflage regional variation across the country’s large and socio-economically diverse territory.

This article aimed to quantify the regional inequalities and impacts of associated risk factors on the prevalence of ARI among pre-schoolers in India. The information extracted from the latest wave of Indian National Family Health Survey (NFHS–4) conducted in 2015–16 was utilised. The novelty of this study is therefore, the use of recent nationally representative samples of NFHS-4 at a disaggregated level to examine the prevalence and risk factors associated with ARI. Regional inequalities in the prevalence of ARI episode were quantified and presented through a map of India and forest plots. Risk factors of childhood ARI were identified using bivariate analyses and multivariable logistic regression model. The study outputs will fill the gaps in the literature by providing empirical evidence of regional inequalities in the prevalence of ARI in contemporary India. The findings may be helpful in developing policies at the regional and national levels to reduce the rate of ARI-related mortality and morbidity.

## Methods

### Description of the Indian National Family Health Survey (NFHS-4)

This study extracted and analysed information from NFHS–4 conducted in 2015–16. The NFHS–4 adopted a two-stage stratified sampling technique to achieve a representative sample of the whole country. The administrative districts were stratified into urban and rural areas to achieve appropriate representation. In addition, six strata were constructed with the slums of major cities (Chennai, Hyderabad, Indore, Kolkata, Meerut and Nagpur). The sampling frame for the first stage was the list of all primary sampling units (PSUs), which are the villages (rural settings) or census enumeration blocks (urban settings) and were achieved through the 2011 census. In the first stage, the PSUs were selected independently from each stratum using the probability proportional to size technique. A sample consisting of 28 586 PSUs (130 from metropolitan slums, 8 397 from urban and 20 059 from rural areas) was selected in this stage. These PSUs (or segments for large PSU) were considered clusters in the second stage sampling. The sampling frame for the second-stage sampling was the list of households within previously selected PSUs (clusters). With a systematic sampling technique, 22 households were randomly selected from each of the previously selected cluster and constituted the final sample. In field visits, 616 346 households were occupied, and finally, 601 509 households were successfully interviewed with a response rate of 98%. From the selected households, 699 686 ever-married women aged between 15 and 49 years were interviewed. The study was conducted on 238 945 children alive at the time of interview and born to the interviewed women within five years preceding the survey. Information regarding the children (gender, nutritional status, morbidity and age), their mother (age, religion, education, caste, parity and access to media) and household status (sources of cooking fuel, wealth and place of residence) were collected as the response of women and gathered through computer-assisted personal interviews. Detail of the sampling procedure can be obtained from the final report of the survey elsewhere (IIPS & ICF, 2017).

### Variables used in the study

The dependent variable of this study is the incidence of childhood ARI, and the information was gathered from the responses of mothers of infants and not through medical examination. The variable, that is, ARI, is dichotomous in nature with various levels of suffering or not suffering from episodes, such as cold, cough, breathing difficulty and fever, within two weeks preceding the survey. A child having illness with a cough accompanied by short, rapid breathing or by difficulty in breathing was identified as an episode. The association of childhood ARI with a number of covariates was quantified and tested.

India is a federal country comprising of twenty-nine states and seven union territories which are categorised into six regions: North (Jammu and Kashmir, Himachal Pradesh, Punjab, Chandigarh, Uttarakhand, Haryana and Delhi), Central (Rajasthan, Uttar Pradesh, Chhattisgarh and Madhya Pradesh), East (West Bengal, Jharkhand, Odisha and Bihar), North-east (Sikkim, Arunachal Pradesh, Nagaland, Manipur, Mizoram, Tripura, Meghalaya and Assam), West (Gujarat, Maharashtra and Goa) and South (Andhra Pradesh, Karnataka, Kerala, Tamil Nadu and Puducherry).

Based on hereditary, occupation and endogamy, a centuries-old social structure has developed in India, called the ‘caste system. The caste system is well documented in the Government of India (Scheduled Castes) Order, 1936 and also in the Constitution (Scheduled Castes) Order, 1950. In that system, the socially disadvantaged groups are identified as ‘scheduled castes’ (SCs) and ‘scheduled tribes’ (STs). Educationally or socially disadvantaged individuals have been classified as ‘other backward castes’ (OBCs) and the ‘forward caste’ (FC) constitutes with the high caste groups (but exclude some upper-class groups). In this study, the ‘Caste’ variable has entered with the levels: SCs, STs, OBCs and forward castes (FCs).

The selection of covariates was confirmed through contemporary literature. Table 1 presents the detailed categorisation of the covariates with percent distribution.

**Table 1:** Percent distribution of children with various background who did and did not suffer from ARI

Variable	Level	Total children	% with ARI
Child related covariates			
Incidence of diarrhoea^***^	Yes	21,920	8.8
	No	217,027	2.1
Anaemia^***^	Severe to moderate	61,785	3.0
	Mild to not anemic	138,745	2.7
Nutritional status (stunting) ^***^	Severe	34,983	3.0
	Moderate	47,715	2.9
	Normal	145,318	2.6
Sex of child^***^	Male	124,493	2.9
	Female	114,452	2.5
Age in month^***^	0–11 months	46,109	3.4
	11–23 months	47,839	3.3
	24–35 months	47,398	2.6
	36–59 months	97,600	2.2
Mother related covariates			
Mother age^***^	15–19 years	6709	3.4
	20–24 years	76,735	2.9
	25–29 years	93,464	2.6
	30–49 years	62,038	2.6
Level of mother’s education^**^	Illiterate	70,636	2.7
	Primary	33,239	3.0
	Secondary	109,445	2.7
	Higher	25,626	2.4
Mother’s religion^***^	Hindu	187,795	2.6
	Muslim	39,564	3.2
	Christian	4969	2.1
	Other	6617	2.7
Mother’s caste^***^	Schedule caste	51,209	3.0
	Schedule tribe	25,052	2.2
	OBC	105,386	2.7
	Other	48,896	2.7
Mother’s Access to electronic media^***^	Have access	170,277	2.7
	No access	68,668	2.9
Total children ever born^***^	One birth	60,423	3.1
	Two births	91,031	2.5
	3 + births	87,490	2.7
Mother’s body mass index (BMI) p > 0.1	Underweight	58,133	2.8
	Normal	141,708	2.7
	Overweight	34,771	2.7
Community and household related covariates			
Source of cooking fuel^***^	Clean	77,899	2.4
	Not clean	146,168	2.9
Wealth index^***^	Poor	111,569	3.0
	Middle	91,390	2.6
	Rich	35,986	2.3
Region^***^	North	31,622	2.9
	Central	63,451	3.8
	East	60,802	2.8
	North-east	8446	1.6
	West	30,903	2.0
	South	43,721	1.7
Altitude	Normal	233,234	2.7
	High	5712	3.0
Place of residence^***^	Urban	67,958	2.3
	Rural	170,987	2.9
Overall			2.7

Significance at ***p < 0.001; **p < 0.05; *p < 0.1

### Statistical analyses

The incidence of ARI among children aged between 0 and 59 months and living across all the states of India were presented using maps. Within-region variations of the incidence were combined and compared using forest plots to visually explore the variability of ARI prevalence among regions. Bivariate and multivariate logistic regression models were used to quantify the association of socioeconomic and demographic characteristics with the prevalence of childhood ARI. Data processing and statistical analyses were conducted using SPSS 26.0 (Zou et al., 2020) and R 3.5.3 (Team, 2019). The sample weight of multi-stage cluster sampling was incorporated in the analytical procedure through R package by survey. Map tools (Bivand & Lewin-Koh, 2019) package in R was used to present the prevalence of ARI in the map of India, whereas the *rmeta* (Lumley & Lumley, 2018) package was used for meta-analysis.

## Results

Subnational level inequalities are evident in the prevalence of ARI for a country with approximately 1.37 billion population and the highest possible socioeconomic and demographic diversities. Based on diversities in cultural settings, socioeconomic achievements and development indicators, contemporary public health research in India segregates the analyses into six geographical regions (Singh, 2013). This broad categorisation was adopted in the current research.

**Fig. 1:**
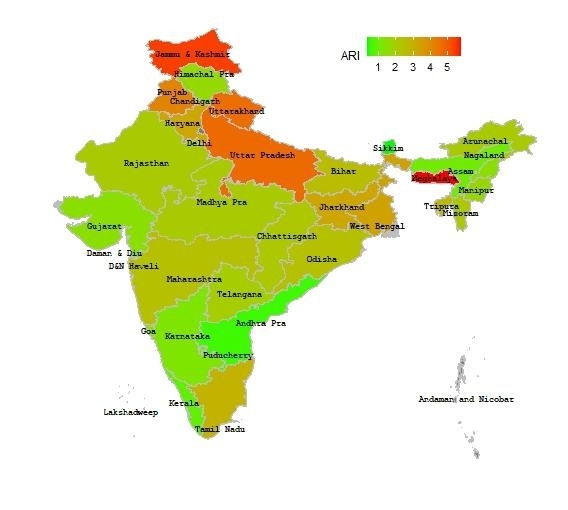
Map of Indian states and union territories showing percentages of children aged below five years and suffering from ARI

The percentages of children aged below five years and suffering from ARI in 29 states and 7 union territories were presented in the map of India (Fig. 1). Children from the states located at the northern region of India showed relatively high prevalence of ARI. Jammu and Kashmir (6.4%), Uttarakhand (4.9%), Uttar Pradesh (4.7%) and Punjab (4.6%) are among the states with relatively high prevalence of ARI. Among the states in the east region, West Bengal had the highest percentage of children suffering from ARI (3.3%). A state in the northeast region of India, Meghalaya (5.8%) had the highest prevalence, whereas Sikkim, Assam and Nagaland exhibited a relatively lower prevalence. The south and west regions of India presented low prevalence of ARI, with the highest prevalence occurring in Tamil Nadu (2.8%).

**Fig. 2:**
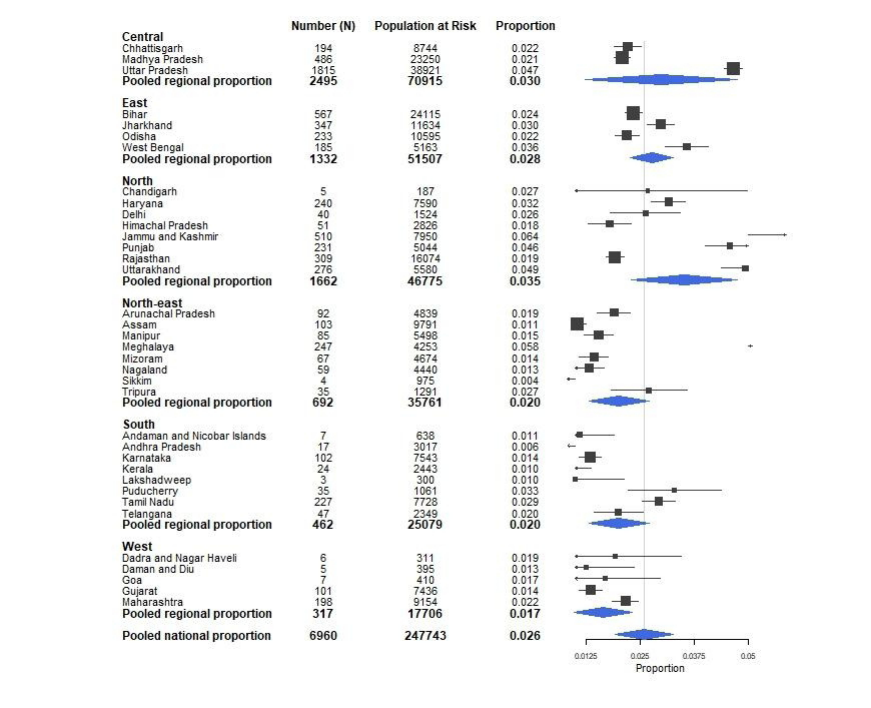
Forest plot representing intra- and inter-regional inequalities in the proportion of children in India suffering from ARI

The forest plot in Fig. 2 presents the inequalities in the prevalence of childhood ARI within and between the regions of India. The prevalence of ARI incidence in each state in Fig. 2 is represented by a square, with a horizontal line indicating the confidence interval, whereas the size of the square reflects the weight of each studied state in the meta-analysis. The vertical line represents the pooled national proportion obtained using meta-analysis. The width of the diamond shows the 95% confidence interval, with the centre representing the pooled national or regional proportion. The pooled estimate of the prevalence of ARI for the states within the west region (1.4%) was significantly lower than the national pooled estimate (2.6%). The states within the region had consistently low estimated prevalence of ARI (between 1.3% and 2.2%). A relatively low confidence band was observed for Gujarat and Maharashtra, with the latter showing a higher prevalence. Except for Tamil Nadu and Puducherry, all states in the south region had significantly lower prevalence than the national average. The ARI rate in all other states in the northeast region had consistently low prevalence.

The pooled prevalence of the incidence of childhood ARI in the northeast region of India was significantly lower than the overall prevalence in the country. However, the incidence was significantly high in Mizoram. As shown in the forest plot, the pooled prevalence of childhood ARI in the north region did not differ significantly from the national average. States in the north region showed the highest variation in the prevalence than any other regions of India. Out of the eight states in the region, the prevalence was significantly higher in four states (Haryana, Jammu and Kashmir, Punjab and Uttarakhand). In the region, the prevalence was significantly low in Himachal Pradesh and Rajasthan. The pooled estimate for the prevalence of childhood ARI in the east region was 2.8%, which did not differ significantly from the national level prevalence. However, the prevalence was significantly high in Jharkhand and West Bengal. For the two other states in the region (Bihar and Odisha), the prevalence showed no significant difference from the national average. The pooled estimate of the prevalence for the three states in the central region showed no significant difference from the national average. The prevalence was significantly higher (4.7%) in Uttar Pradesh, significantly lower in the Madhya Pradesh and exhibited no significant difference in Chhattrisgarh.

The incidence of diarrhoea, anaemia, stunting, gender and age of children were significantly associated with childhood ARI. For instance, the prevalence of ARI for the children suffering from diarrhoea was 8.8%. The percentage was 2.1% for those who were not suffering from diarrhoea within two weeks preceding the survey. Among those suffering from severe anaemia, the percentage of ARI was 3.7%, whereas the percentage was 2.6% for those who were not suffering from anaemia. Severely stunted children were more likely to suffer from ARI than those who were not stunted. Older children (aged 36–59 months) were less likely (2.2%) to suffer from ARI than the younger (aged less than one year) cohort (3.4%).

Children with teenage women were more likely (3.4%) to suffer from ARI than those whose mothers were aged between 25 and 49 years. No specific association was observed between the prevalence of ARI of children and educational attainment of mothers. Children of Muslim women were more likely (3.2%) to suffer from ARI than those whose mothers have other religious affiliation. The incidence of childhood ARI was significantly high among the children with schedule caste background. Children from households using unclean cooking fuel were significantly more likely to suffer from ARI (2.9%) than those from households using clean cooking fuel. Children from poor households were more likely (3.0%) to suffer from ARI than those from rich households (2.3%).

The incidence of childhood ARI was significantly lower in northeast (1.6%) and south (1.7%) regions and significantly higher in the central (3.8%) regions of India. Children living at high altitude were more likely to suffer from ARI than the others. However, the difference was not statistically significant. Children from rural areas were significantly more likely (2.9%) to suffer from ARI than those from their urban counterparts (2.3%).

The associations of childhood ARI (aged below five years) with a set of background characteristics were identified using a multivariable logistic regression model. Among the variables representing background characteristics of children, the incidence of diarrhoea, age and gender showed significant association with ARI. Religious affiliation, caste and parity of women were also significantly associated with the incidence of ARI of children. Significant variations in the prevalence of ARI were observed across Indian regions (Table 2).

**Table 2:** Results of binary logistic regression model for ARI prevalence for under 5 children in India

Variable	Levels	AOR (CI)
Incidence of Diarrhoea	No	1.00
	Yes	3.75 (3.40 to 4.13)^***^
Sex of child	Female	1.00
	Male	1.18 (1.09 to 1.27) ^***^
Nutrition status of child	Normal	1.00
	Moderate Stunted	1.08 (0.97to 1.21)
	Severe Stunted	1.05 (0.93to 1.19)
Anaemia	Mild or not anaemic	1.00
	Severe-Moderate	0.95 (0.87to1.03)
Age of child	36 to 59 months	1.00
	0–11	1.34 (1.18 to 1.53) ^***^
	12–23	1.17 (1.05 to 1.31) ^***^
	24–35	1.09 (0.98 to 1.22)
Age of mother	30 to 49 years	1.00
	15–19	0.77 (0.57 to 1.05)
	20–24	0.99 (0.87 to 1.13)
	25–29	0.97 (0.87 to 1.08)
Religion of mother	Christian	1.00
	Hindu	0.72 (0.52 to 0.98) ^**^
	Muslim	0.89 (0.64 to 1.24)
	Other	0.80 (0.54 to 1.19)
Caste of mother	Forward caste	1.00
	Schedule tribe	0.80 (0.66 to 0.97) ^***^
	Schedule caste	1.05 (0.92 to 1.21)
	Other backward class (OBC)	0.93 (0.83 to 1.05)
Mother’s access to Electronic media	Having access	1.00
	No access	0.94 (0.85 to 1.04)
Mother’s total children ever born	Three or more births	
	One birth	1.21 (1.06 to 1.40) ^***^
	Two births	1.00 ( 0.90 to 1.11)
Mother’s nutrition status	BMI Normal	1.00
	Underweight	0.98 ( 0.89 to 1.08)
	Overweight	1.16 (1.00 to 1.33)
Mother’s education	Higher education	1.00
	Illiterate	0.84 (0.69 to 1.01)
	Primary	0.99 (0.81 to 1.21)
	Secondary	1.00 (0.85 to 1.19)
Place of residence	Urban	1.00
	Rural	1.11 (0.97 to 1.28)
Wealth	Rich	1.00
	Poor	1.22 (1.00 to 1.51) ^*^
	Middle	1.12 (0.93 to 1.35)
Region	Ref: North-East	1.00
	North	1.68 (1.38 to 2.06) ^***^
	Central	2.02 (1.67 to 2.43) ^***^
	East	1.57 (1.28 to 1.92) ^***^
	West	1.31 (0.99 to 1.74) ^*^
	South	0.97 (0.77 to 1.21)

Significance at ***p < 0.001; **p < 0.05; *p < 0.1

Childhood co-morbidity was identified, that is, those who were suffering from diarrhoea were 2.75 times more likely to be exposed to ARI than those who were not suffering from such condition (adjusted odds ratio [AOR]: 3.75; confidence interval (CI): 3.40 to 4.13). Male children were 18% (AOR: 1.18; CI: 1.09 to 1.27) more likely to suffer from ARI than female children. The model considered children aged between 36 and 59 months as reference category. In comparison with the reference category, those aged below 11 months (AOR: 1.34; CI: 1.18 to 1.53) and those aged between 12 and 23 months (AOR: 1.17; CI: 1.05 to 1.31) were 34% and 17% more likely to suffer from ARI, respectively. After adjusting for other explanatory variables, age, education or access to electronic media of women showed no significant association with the incidence of ARI of their children. On the other hand, children whose mothers have Hindu religious background and from schedule tribes were significantly less likely to suffer from ARI. Children from mothers who gave birth once possessed 21% (AOR: 1.21; CI: 1.06–1.40) higher risk of suffering from ARI compared with those whose mother had 3 or more births. Urban–rural residence or wealth status of household showed no significant association with childhood ARI. In the multivariable analysis, the northeast region was considered the reference. With respect to the reference category, children from the north, central and east regions were 0.68 (AOR: 1.68; CI: 1.38–2.06), 1.02 (AOR: 2.02; CI: 1.97–2.43) and 0.57 (AOR: 1.57; CI: 1.28–1.92) times more likely to suffer from ARI than the others, respectively.

## Discussion

This study is based on the information collected from the latest wave of the largest demographic and health survey data (NFHS-4) in India. Through mapping and meta-analyses, the study quantified regional inequalities in the incidence of childhood ARI in India. The degree of associations of socioeconomic, cultural and geographic variables of ARI were quantified using binary and multivariable analyses.

Socio-economic and environmental differences across the states and territories of India are evident in contemporary literature (Awasthi et al., 2018; Chakraborty & Ghosh, 2019; Gothankar et al., 2018; Mareeswaran et al., 2018). As observed in the result of this study, the differentials are transferred into the incidence of childhood morbidity. Differences in environmental quality, including air pollution and density of population, may be considered to form the uniqueness of a region in India regarding the prevalence of ARI among children under five years. Similar findings were reported in several studies conducted in rural parts of India (Bhat & Manjunath, 2013; Choube et al., 2014; Ladomenou et al., 2010; Sharma et al., 2013; Smith et al., 2000; Taksande & Yeole, 2015). According to the binary logistic regression analysis, those suffering from diarrhoea possess relatively high risk of suffering from ARI, although this relationship may work in the opposite manner. A similar interrelationship was observed in other studies in South Asian region (Hasan & Richardson, 2017; Mareeswaran et al., 2018). In line with other studies (Choube et al., 2014; Goel et al., 2012; Prajapati et al., 2012), this research confirms that male children are more vulnerable to ARI than their female counterparts. A possible reason may be the comparatively higher outgoing movements and weaker immune system and ‘male disadvantage’ of boys, which render them more susceptible to acquire respiratory infections from ambient air pollution than girls (Giefing-Kröll et al., 2015; Markle & Fish, 2014). This study identified significant age inequities in the incidence of childhood ARI, in which older children are considered more protected. Similar outputs have been observed in a number of studies conducted at the local (states across India), national and global levels (Acharya et al., 2003; Islam et al., 2013; Kumar et al., 2015; Mitra, 2001; Monto & Ullman, 1974; Prajapati et al., 2011; Reddaiah & Kapoor, 1990; Tupasi et al., 1988). Although other studies found insignificant association between age and the incidence of childhood ARI (Reddy et al., 2016; Sharma et al., 2013; Tazinya et al., 2018), the relatively high prevalence of ARI among 2–3-year-old children can be due to their high exposure to environmental factors.

The findings of this study may positively influence the ever-growing health issues of ARI among under-five children in India. At the same time, our study has several limitations. Firstly, the incidence of ARIs was collected from the reports of women without any medical validation. Secondly, the variables were self-reported and might suffer from reporting error or recall bias. Thirdly, we did not consider any data on whether the children have any pre-existing illness that might cause the symptoms of ARI. Fourthly, the data were drawn from secondary sources and may be inappropriate for any causal inferences. Lastly, given the cross-sectional nature of the study, a cause-effect relationship could not be demonstrated. Despite these limitations, our study has a number of strengths. This study used nationally representative data. Thus, results can be generalised at the national level. This research has implications for policy making and interventions at the national and sub-national levels. India’s states and territories vary widely in terms of their natural resource endowments, socio-economic structures and climate conditions. Most of the health care and healthcare delivery decisions and resource allocation planning are made and implemented at the state level. Childhood mortality and morbidity attributable to ARI must be assessed at the national and regional levels to inform policies and strategies and measure progress towards achieving child health outcomes of SDGs. This study adds value to the better understanding of inequality patterns and quantifies within- and intra-region inequalities of ARI prevalence in India.

## Conclusions

With empirical evidence, this research highlighted the regional variation and risk factors of ARI in India. The statistical analyses were conducted to test the association between ARI and several risk factors. Education, age, place of residence and access to electronic media of mothers, wealth status of household, and anaemia and nutritional status of children were not significantly associated with ARI. Children’s sex, age and incidence of diarrhoea had significant association with ARI. Specific groups of children with higher prevalence of ARI include those living in central region, aged between 0 and 23 months and suffering from diarrhoea. Therefore, these important risk factors of ARI must be addressed to prevent the associated morbidity and mortality and fulfil the SDG on children’s health.

## Data Availability

The data that support the findings of this study are openly available in National Family Health Survey at https://www.dhsprogram.com/.
